# Biofortification, Crop Adoption and Health Information: Impact Pathways in Mozambique and Uganda

**DOI:** 10.1093/ajae/aay005

**Published:** 2018-03-15

**Authors:** Alan de Brauw, Patrick Eozenou, Daniel O. Gilligan, Christine Hotz, Neha Kumar, J.V. Meenakshi

**Keywords:** Biofortification, technology adoption, Mozambique, Uganda, casual mediation analysis, randomized control trial

## Abstract

Biofortification is a promising strategy to combat micronutrient malnutrition by promoting the adoption of staple food crops bred to be dense sources of specific micronutrients. Research on biofortified orange-fleshed sweet potato (OFSP) has shown that the crop improves the vitamin A status of children who consume as little as 100 grams per day, and intensive promotion strategies improve dietary intakes of vitamin A in field experiments. However, little is known about OFSP adoption behavior, or about the role that nutrition information plays in promoting adoption and changing diet. We report evidence from similar randomized field experiments conducted in Mozambique and Uganda to promote OFSP. We further use causal mediation analysis to study impact pathways for adoption and dietary intakes. Despite different agronomic conditions and sweet potato cropping patterns across the two countries, the project had similar impacts, leading to adoption by 61% to 68% of farmers exposed to the project, and doubling vitamin A intakes in children. In both countries, two intervention models that differed in training intensity and cost had comparable impacts relative to the control group. The project increased the knowledge of key nutrition messages; however, added knowledge of nutrition messages appears to have minimally affected adoption, conditional on assumptions required for causal mediation analysis. Increased vitamin A intakes were largely explained by adoption and not by nutrition knowledge gained, though in Uganda a large share of impacts on vitamin A intakes cannot be explained by mediating variables. Similar impacts could likely have been achieved by reducing the scope of nutrition trainings.

*JEL codes:* I15, O12, O13, Q12.

Micronutrient malnutrition continues to be a major health problem affecting developing countries, and in Sub-Saharan Africa (SSA) in particular. Indeed, such malnutrition is responsible for a significant share of infant mortality (Bryce et al. 2003) and hinders human capital development (Alderman, Hoddinott, and Kinsey 2006). Vitamin A deficiency (VAD) is a leading form of micronutrient malnutrition, and is an important cause of morbidity, impaired night vision and, in more severe manifestations, of blindness and increased mortality in young children. VAD affects nearly 127 million pre-school aged children worldwide and accounts for 6% of all deaths among children under 5 years of age (Fawzi et al. 1994; Villamor and Fawzi 2000; West, Jr. 2002; Black et al. 2008). Aguayo and Baker (2005) argue that “…effective and sustained control of (VAD) has the potential to be among the most cost-effective and high-impact child-survival interventions in Sub-Saharan Africa.” In Mozambique and Uganda, the countries selected for this study, 69% and 28% of preschool children are vitamin A deficient, respectively (Uganda Bureau of Statistics and ORC Macro 2001; Aguayo and Baker 2005). VAD disorders also affect adult women by increasing morbidity and mortality during pregnancy (West et al. 1999; Christian et al. 2000).

Biofortification, which entails breeding staple food crops for substantially improved micronutrient content, is an emerging strategy to alleviate micronutrient malnutrition, including VAD. If biofortified staples are available, consumers can substitute low nutrient staples with nutrient-dense varieties of the same crops (Bouis 2002). As an agricultural intervention to address micronutrient deficiencies, biofortification has several advantages. First, staples are consumed daily and constitute a large proportion of diets of poor households, making biofortification pro-poor. Second, once the biofortified variety has been widely adopted, with good access to planting material, the crop can be grown and consumed for years at a low recurring cost. Third, biofortification has the potential to reach vulnerable populations in remote areas that do not have access to commercially-marketed fortified foods and that are more likely to be missed by public supplementation campaigns. Finally, biofortified varieties are selected for their high yields prior to release. The expectation is that absent well-developed markets, increased production from growing biofortified varieties would translate into increased (local) consumption of these nutrient-rich foods.

Before biofortified crops can play a role in improving public health in SSA, farmers must first adopt them. Biofortified crops face many of the constraints that limit adoption of other agricultural technologies in SSA, as well as some new challenges and opportunities. Smallholder adoption will not occur unless farmers expect benefits of adoption, whether monetary or not, to exceed those of their present practices (Foster and Rosenzweig 2010; Jack 2011). Heterogeneity in expected returns may also limit the pool of adopters (Suri 2011). And smallholders may lack information about how to grow biofortified crops or their potential yields (e.g., Almekinders and Hardon 2006). If national seed systems are weak, uptake may lag even if information on the benefits of the crop is available. A unique challenge for biofortified crops is that adoption strategies rely on farmers replacing a low-nutrient staple food crop with high-nutrient varieties, which have some differences in agronomic characteristics. Perceived or actual production risks may increase if farmers begin to substitute away from established crops.

These concerns motivated the intervention design of the HarvestPlus Reaching End Users (REU) project, which distributed provitamin A-rich orange-fleshed sweet potato (OFSP) in Mozambique and Uganda to increase dietary vitamin A intakes in the local population.^1^ To meet this goal, the REU conducted an integrated program to both encourage adoption of OFSP and improve knowledge of the benefits of vitamin A, and thereby increase OFSP consumption, particularly by women and children. Achieving high rates of OFSP adoption and expanding planted area was essential, as most households consumed sweet potato from their own production. OFSP production could be increased directly by providing vines and agronomic training, as well as creating demand for OFSP vines through the nutrition knowledge component and relaxing any information constraints about the benefits of OFSP consumption.

A distinguishing feature of the REU is that it ran very similar programs in both Mozambique and Uganda with broadly common features, including seed systems (production) and demand creation (nutrition) components. In many respects, Uganda represents an ideal context for testing the introduction of OFSP: sweet potato (white- or yellow-fleshed) is the primary staple crop in the study region, so increased access to dietary vitamin A can be achieved by substituting OFSP for low-nutrient sweet potato varieties in production and consumption without other changes in cropping patterns or farm area expansion. In Mozambique, on the other hand, sweet potato is a secondary staple, so partial substitution of sweet potato area into OFSP might provide only limited increases in vitamin A intake. At the outset, it was unclear whether successful adoption of OFSP in Mozambique would require farm area expansion or potentially reduced production of other nutrient dense crops. In order to learn about the impact and cost-effectiveness of the biofortification strategy in these two very different settings, the project incorporated cluster randomized-control trials in each country with three arms: two treatments, and a control group. The intensive treatment (IT) arm included OFSP vine distribution and agriculture and nutrition training over two years, while the moderate treatment arm (MT) was identical to IT in year 1, but included no trainings in year 2 to reduce costs. The primary outcomes studied after the intervention ended were adoption, nutrition knowledge, and vitamin A intakes. In both countries, coordinated baseline and endline surveys were conducted to measure outcomes in a comparable way across the two countries while capturing differences in contexts.

Previous research from this project (Hotz et al. 2012a,b) showed that the REU succeeded in improving dietary intakes of both OFSP and vitamin A among target groups, as well as reducing the prevalence of inadequate vitamin A intakes.^2^ This article builds on that evidence by analyzing impacts on adoption and nutrition knowledge, as well as impact pathways to adoption and vitamin A intakes. First, we examine the impact of the REU on OFSP adoption and nutrition knowledge in both countries, and examine whether impacts vary by intervention model. We exploit differences in the two countries to consider how OFSP adoption behavior varies with initial cropping and dietary patterns. Second, we use causal mediation analysis to quantify the contributions of nutrition knowledge and OFSP adoption to improved vitamin A intakes (Imai, Keele, and Yamamoto 2011). Finally, we discuss the cost effectiveness of the REU, drawing implications for the design of cost-effective scaled-up interventions to disseminate OFSP.

The article makes several contributions to the literature. First, it provides experimental estimates of adoption behavior of households exposed to a biofortified staple food crop, showing that roughly 2 out of 3 households in two countries undertake sustained adoption of the crop over multiple seasons. These results are relevant to the growing literature on constraints to the adoption of agricultural technologies. Second, it contributes to the growing literature on major pathways to impact using causal mediation analysis, providing insights on the role of information in agriculture-nutrition interventions, as well as the importance of household access to nutritious foods in improving nutritional outcomes. Third, it provides evidence of impact in two countries that differ in important respects. For example, sweet potato is the primary staple in Uganda—and constitutes a larger part of the diet—than in Mozambique, where it is a secondary crop. Growing seasons, and the ability to maintain sweet potato vines across seasons, also vary greatly in the two countries. Therefore, it is plausible that both impacts and the impact pathways vary significantly across the two countries. Finally, unlike much of the literature, we address the cost effectiveness of implementing the REU. In so doing, we explicitly measure the extent to which OFSP spread to households beyond targeted beneficiaries in both countries.

## Evaluating the REU in Mozambique and Uganda

In this section, we first describe the contextual differences between the two countries, then briefly explain how the REU was implemented, and how samples were constructed in both countries. Finally, we discuss pertinent components of the data collected for the evaluation.

### Contextual Differences: Mozambique and Uganda

Northern Mozambique and Central/Eastern Uganda were two very different contexts in which to conduct a biofortification experiment through the introduction of OFSP. Sweet potatoes are the primary staple food crop in this part of Uganda, accounting for 16% to 18% of cultivated area at baseline. In northern Mozambique, on the other hand, sweet potatoes are one of several secondary staples, occupying only 8% of its cultivated area at baseline. Mozambique also only has one rainy season, and therefore most agricultural production takes place over one season. In Uganda, there are two productive seasons, making it easier for farmers to maintain their own source of OFSP vines between seasons. As a result of these differences, the REU project implemented different dissemination strategies across the two countries, delivering OFSP vines to project farmers annually in Mozambique but only once in Uganda, while the models of training intensity for extension and nutrition were kept the same. These differences in context are borne out in descriptive statistics shown in table 1 on the proportion of households growing sweet potatoes at baseline and the average area sown in sweet potatoes. As such, the objective in Mozambique was largely to attract farmers to grow OFSP, whereas the objective was to induce farmers to substitute white or yellow sweet potato for OFSP in Uganda. In addition, patterns of dietary intake of vitamin A differed across the two countries. In Mozambique, the prevalence of inadequate vitamin A intakes was 80% to 84% among women of child-bearing age at baseline, whereas it was between 28% and 43% in Uganda.

**Table 1:** Baseline Household Descriptive Statistics, by Model, REU, Mozambique and Uganda

	Mozambique			p-value, IT= MT=C	Uganda			p-value, IT= MT=C
	Intensive Treatment	Moderate Treatment	Control		Intensive Treatment	Moderate Treatment	Control	
**Household Characteristics**								
Female head	0.05	0.07	0.07	0.642	0.11	0.19	0.12	0.035
Household size	5.82 (1.94)	5.81 (1.81)	5.84 (1.80)	0.977	7.68 (2.87)	7.39 (2.59)	7.79 (2.94)	0.359
Years of schooling, head	2.74 (2.49)	3.77 (2.62)	2.88 (2.39)	0.078	6.87 (3.49)	7.31 (3.74)	7.31 (3.78)	0.203
Log, monthly per capita expenditures	0.89 (0.71)	1.05 (0.70)	0.98 (0.79)	0.273	10.00 (0.75)	9.98 (0.75)	10.03 (0.69)	0.744
Leader or promoter in household?	0.21	0.24	N/A		0.17	0.17	0.21	0.400
**Production Characteristics**								
Grew any sweet potato in year before baseline (1=yes)	0.47	0.55	0.51	0.604	0.85	0.83	0.87	0.403
Total Area (acres), sweet potato, year before baseline	0.31 (0.63)	0.42 (0.83)	0.26 (0.56)	0.294	0.28 (0.36)	0.24 (0.28)	0.29 (0.50)	0.487
Access to lowlands (1=yes)	0.62	0.65	0.66	0.931	0.43	0.38	0.42	0.538
Grew OFSP prior to baseline (1=yes)	0.11	0.09	0.06	0.480	0.08	0.05	0.07	0.359

*Note:* Standard deviations appear in parentheses for continuous variables. There are 628 observations for Mozambique and 975 observations for Uganda. Reference children in Uganda were between age 3 and 5 at baseline, hence they were no longer breastfed.

*Source:* REU Baseline and Endline Survey Data, Mozambique and Uganda.

### REU Implementation

The REU intervention built on earlier work demonstrating that OFSP intakes improved vitamin A status (e.g., Hagenimana et al. 2001; van Jaarsveld et al. 2005; Low et al. 2007) and that it would potentially find wide consumer acceptance (e.g., Naico and Lusk 2010; Chowdhury et al. 2011). However, these studies share two deficiencies. First, they did not study adoption behavior, and second, they did not provide any evidence on cost effectiveness of the OFSP-based dissemination strategies.

The REU intervention design was integrated across two primary components. The first consisted of seed systems and extension and focused on OFSP production, in which the project distributed vines to farmers and trained them in optimal agronomic practices. The second component consisted of demand creation, which used multiple strategies to inform people about the nutritional benefits of consuming OFSP and vitamin A. While the overall structure of these two components of the intervention were similar across countries, there were some differences that were necessitated on account of varying local conditions (Arimond et al. 2008, 2009).

These components were delivered, as noted earlier, using two treatment strategies that varied in timing and intensity of activities, such that they had different average and marginal costs per beneficiary. In year one of the intervention, the intensive treatment (IT) and moderate treatment (MT) were identical in agricultural extension and nutrition education activities. Differences between the two treatment strategies occurred in year two. In IT, the high intensity of extension visits and nutrition messages from year one were continued in year two. In MT, the activities in agriculture and nutrition messaging were substantially scaled back in the second year to provide cost savings. In year one, the same treatment intensity was used because initially intense activity was considered necessary for the crop to be adopted and accepted. In both countries, multiple OFSP varieties were distributed during vine dissemination and farmers were trained about the agronomic, taste, and health characteristics of the different varieties. Farmers therefore had the opportunity to try different varieties and determine which ones they preferred to grow and consume.

### Sample Design for Evaluation

The sample size was based on separate power calculations for primary outcomes for each of the two countries.^3^ In both countries, based on the calculated necessary sample sizes, the goal was to interview exactly the same set of households and reference children in the baseline and endline surveys.^4^

The Mozambique sample is composed of 36 community organizatons, each in a separate village, from Milange, Gurué, Nicoadala, and Mopeia districts of Zambézia Province. Twelve community organizations were assigned to IT, MT, and a control group, respectively, stratified by district.^5^ Power calculations indicated that 12 households per community organization be included in the nutrition survey to identify an effect on vitamin A intakes half the magnitude of that found in Low et al. (2007) at the 5% level with 80% power. Given additional returns to collecting socioeconomic data and adoption data indicated by power calculations on general discrete variables, the goal was to conduct the socioeconomic survey in 20 households per community organization.

Communities that were initially selected had to meet four salient requirements: first, they had to have enough families with resident children between the ages of 6 and 35 months at baseline to be able to meet sample size requirements; second, they had to have reasonable access to lowlands so that vines could be kept between growing seasons; third, other agricultural interventions were not active in selected communities, and selected communities had not been previously targeted for an OFSP project; fourth, the selected communities could not be adjacent to one another, to minimize contamination and jealousy between communities.^6^ The 36 villages included in the sample were then randomly selected into one of the two treatment arms or the control group, stratified by district. A total of 703 households were included in the socioeconomic survey baseline sample; in the endline survey, 628 households were resurveyed.^7^

The Uganda sample includes 84 farmer groups from three districts: Kamuli, Bukedea, and Mukono, which were selected for the project because white- and yellow-fleshed sweet potato is commonly grown and consumed there, and the districts are relatively close to potential markets for OFSP. Farmer groups were sampled from a list of active farmer groups in each district obtained from NGO implementing partners based on consultation with local leaders. Within district strata, farmer groups were randomly assigned into one of two treatment arms (IT and MT) or the control group. The Uganda sample is unbalanced, including 36 farmer groups in IT, 12 in MT, and 36 in the control group, as serum retinol collected in IT and the control group required larger sample sizes to attain minimum detectable effects (see Hotz et al. 2012b for more information). In each farmer group, 14 households were selected so the baseline sample is 1,176 farmer group member households; of these households, 1,116 households were also surveyed at endline.^8^

In contrast to Mozambique, in Uganda reference children were defined as children aged 3 to 5 years of age (36 to 71 months), so that nearly all of these children would age out of the Ugandan government’s vitamin A supplementation program a few months before the endline survey. Dietary intakes were collected in households of all farmer groups but the sampling of reference children for this purpose was unbalanced in order to account for the smaller number of clusters in IT. In IT and control clusters, eight reference children aged 3 to 5 years were randomly selected from sample households, while in MT clusters, 14 children were selected for dietary intake interviews. This procedure resulted in 576 reference children in the baseline.

### Data Collection Survey Content

Baseline socioeconomic surveys were conducted in 2006 in Mozambique, and in 2007 in Uganda. The questionnaires in each country were similar, but modified for relevance to the local context. Descriptive statistics on baseline household characteristics at baseline between IT, MT, and control show few statistically significant differences between averages across the three groups (see table 1).

The endline surveys conducted in 2009 in both countries largely followed the structure of the baseline surveys, but included redesigned modules related to sweet potato production and consumption to learn specific details about the experience that households had in growing OFSP during the REU. We asked about production since the project began; due to concerns regarding potential recall bias, we asked a more detailed set of questions about the previous 12 months and more limited questions about prior seasons.

Using this information, we measure adoption (*A_i_*) in three ways: first, as an indicator variable, defined as whether farmers kept vines for the following season (Mozambique), or if farmers were growing OFSP at the time of the final survey (Uganda).^9^ Second, we measured the intensity of adoption by the share of OFSP in the total sweet potato area farmed by the household. The drawback to this variable is that it is undefined for households that do not grow sweet potatoes; yet for those that do grow sweet potatoes, it measures the commitment to OFSP quite well. Third, we use the total area under OFSP cultivation.^10^

An important survey component dealt with nutrition knowledge. Surveys asked a detailed set of questions of female caregivers, including open-ended questions about the role of vitamin A in the diet and vitamin A sources. Enumerators then were asked to mark any correct answers in the former case, and to check off foods from a list in the latter. Responses are used to define two measures of nutrition knowledge (*N_i_*): the number of facts about vitamin A promoted by the REU that mothers could recite, and conditional on knowing about vitamin A, whether mothers named OFSP as a vitamin A source.

Finally, in both countries a module was included to understand whether households either gave or received OFSP planting material to other households within or outside their farmer groups. The module was used to construct an estimate of the additional households that were able to grow OFSP as a result of the program. The data were cleaned and compared to beneficiary lists to ensure that direct beneficiaries were not also included in our estimates of indirect beneficiaries, which are used to compute cost effectiveness.

In both countries, baseline nutrition surveys took place alongside the socioeconomic surveys. In the endline, the nutrition surveys took place in advance of the endline socioeconomic survey, so that OFSP would still be in the field and be consumed by households. As with the socioeconomic survey, the endline survey had to identify the correct reference child in each of the panel households. The main component of this survey was devoted to capturing the primary nutrition outcome variable, child-specific vitamin A intakes (*V_i_*). It used an interactive, multiple-pass method to measure individual-specific food intakes in the 24-hour period ending on the morning of the interview. Additional details on the procedure followed to compute *V_i_* can be found in Arimond et al. (2009) and Hotz et al. (2012).^11^

## Conceptual Framework and Estimation Strategy

A useful way to conceptualize how the introduction of OFSP could induce OFSP adoption, eventually reducing VAD, is to begin with an agricultural household model (Singh, Squire, and Strauss 1986).^12^ In the traditional agricultural household production model, the introduction of OFSP can be viewed as a new production technology (akin to a switch from traditional to high-yielding variety of any crop). In turn, the technology would translate into increased intake of OFSP and therefore of vitamin A, absent substitution effects in consumption.

To grow OFSP, farmers could either substitute existing land away from other crops or bring additional area under cultivation. Conditional on adoption, there could be positive or negative income effects, which then could in turn lead to either enhanced or decreased consumption of OFSP and vitamin A-rich foods in general. Furthermore, augmented OFSP availability within the household does not necessarily translate to enhanced consumption among children and women, (even though the nutrition component of the REU specifically targeted messages about OFSP to these groups). Therefore, one channel we test is whether OFSP adoption directly affects vitamin A intakes.

But when—as is often the case in developing countries—some markets are missing (implying non-separability), consumption could also drive production decisions (e.g., Benjamin 1992; LaFave and Thomas 2016). For example, if a household prefers to consume a crop for which a market does not exist, knowledge of the health benefits of OFSP—essentially a change in the consumer information set—could determine whether and how much to plant of OFSP, which in turn would impact vitamin A intakes. Thus, imparting nutrition knowledge, which was the goal of nutrition extensionists, could influence adoption. Nutrition knowledge could also have directly affected vitamin A intakes (through consumption of other vitamin A rich foods, for example).

The above discussion provides the basis for the choice of outcome and mediating variables. The principal mechanisms by which the REU could affect vitamin A intakes are as follows. First, the intervention may translate into better knowledge about the nutritional content of OFSP and of vitamin A in general, thereby increasing OFSP adoption, and in turn vitamin A intakes among children. Second, farmers could simply prefer OFSP varieties to other sweet potatoes or other crops, and plant and consume them without much influence from additional vitamin A knowledge. Third, the information campaign may also affect vitamin A intakes directly, either through market purchases, or by targeting young children as consumers of OFSP or other vitamin A rich foods within the household.^13^

For each primary outcome related to OFSP adoption *A_i_*, nutrition knowledge *N_i_* or vitamin A intakes *V_i_*, the impacts of IT (*T*_1_) and MT (*T*_2_) on outcome *Y_i_*∈{*A_i_, N_i_, V_i_*} among household or child *i* at endline (period 1) can be estimated as

(1) $$\begin{array}{l} Y_{i1}=\alpha +\beta_{1}T_{1i}+\beta_{2}T_{2i}+\gamma X_{i0}+\psi Y_{i0}\\ \;+\varepsilon_{i} \end{array}$$

where *X*_*i*0_ is a vector of baseline household characteristics, *Y*_*i*0_ is the baseline outcome, which is available for nutrition knowledge outcomes, and *ε_i_* is a mean zero error term. Equation (1) is more flexible than the difference-in-differences estimator, as the two are identical if *ψ* is restricted to 1, and has more statistical power than difference-in-differences if the outcome exhibits autocorrelation (McKenzie 2011). Since *X*_*i*0_ and *Y*_*i*0_ are assumed orthogonal to the treatment variable, it is possible to omit them from models with no consequences for the point estimate of *β*. Further, *X*_*i*0_ is primarily included to explain some of the variation in the endline outcome *Y*_*i*1_, reducing overall estimator variance.

The coefficients *β*_1_ and *β*_2_ represent the average intent-to-treat effect on IT and MT households or individuals, respectively. Equation (1) can be used to test whether the intent-to-treat effect is larger than zero for each group, as well as the null hypothesis *β*_1_ = *β*_2_, implying that the impacts of IT and MT were the same. If so, we can instead estimate a simplified model,

(2) $$Y_{i1}=\alpha +\beta T_{i}+\gamma X_{i0}+\psi Y_{i0}+\varepsilon_{i}$$

where *T* now indicates a treatment indicator variable. In estimation, we find very few significant differences between impacts among IT and MT farmers, so we conduct the causal mediation analysis using equation (2) as the primary regression.

### Causal Mediation Analysis

We are also interested in understanding the contribution of additional nutritional knowledge to adoption. As the treatment was randomly assigned, the average treatment effect is identified, but we have to make further assumptions to calculate the average effect of the treatment occurring through a mediating variable *M_i_*(*T_i_*) itself affected by the treatment. Following Imai et al. (2011), we write the causal mediating effect as

(3) $$\delta_{i}(t)\equiv Y_{i}(t,M_{i}(1))-Y_{i}(t,M_{i}(0))$$

for each treatment status *t* = 0, 1. The change in the outcome *Y* corresponding to the change in the mediator variable from the control to the treatment condition while holding the effect of the treatment otherwise constant is represented by *δ_i_*(*t*). For observations receiving the treatment, *M_i_*(0) cannot be observed, so this quantity must be estimated.

The direct effect *ζ_i_*(*t*) of the treatment is what remains after the indirect effect is estimated, and can be written as

(4) $$\zeta_{i}(t)\equiv Y_{i}(1,M_{i}(t))-Y_{i}(0,M_{i}(t)).$$

Averaging over all individuals *i*, the average causal mediation effect (ACME) is $\bar{\delta }(t)$ and the average direct effect (ADE) is $\bar{\zeta }(t)$. The average treatment effect is the sum of the ACME and the ADE $\left(\bar{\delta }(t)+\bar{\zeta }(t)\right)$.

To estimate the ACME and the ADE, we make two assumptions. We first assume that given the baseline characteristics, treatment assignment is independent of outcomes and mediator variables; this assumption holds due to randomization. Second, we make an assumption that Imai, Keele, and Yamamoto (2010) call sequential ignorability:

(5) $$Y_{i}(t,m)⊥M_{i}(t)|T_{i}=t,X_{i}=x.$$

Equation (5) suggests a very strong assumption: once we control for actual treatment status and observed baseline characteristics, the mediator variable is statistically independent of the potential outcome. Clearly, if any unobservable affects both the mediating variable and the outcome, then estimates of the ACME are likely to be biased. However, in exchange for making this strong assumption, we can estimate the ACME and the ADE without additional assumptions (Imai, Keele, and Yamamoto 2010). Further, the method allows us to test the sensitivity of our estimates to unobservables that might be correlated with both the mediator and the outcome. In other words, we test the robustness of our estimates to the assumption in equation (5) to ensure the results do not rely on it.

After making the sequential ignorability assumption, we assume a linear relationship and estimate a set of equations including equation (2) and

(6) $$M_{i1}=\alpha_{2}+\beta_{2}T_{i}+\gamma_{2}X_{i}+\psi_{2}M_{i0}+\varepsilon_{2i}$$

(7) $$\begin{array}{l} Y_{i1}=\alpha_{3}+\beta_{3}T_{i}+\xi M_{i}+\gamma_{3}X_{i}+\psi_{3}Y_{i0}\\ +\varepsilon_{3i}. \end{array}$$

The ACME can be calculated as ${\hat{\beta }}_{2}\hat{\xi }$, where *β*_2_ is the effect of the treatment on the mediator and *ξ* is the effect of the mediator on the outcome. Sequential ignorability implies zero correlation between the error terms *ε*_2*i*_ and *ε*_3*i*_. In this framework, ${\hat{\beta }}_{1}$ and ${\hat{\beta }}_{3}$ are estimates of the ATE and the ADE, respectively.

Imai, Keele, and Yamamoto (2010) further develop a potential sensitivity test for the sequential ignorability assumption. Define *ρ* = *ε*_2*i*_*ε*_3*i*_, or the correlation between the two error terms. If *ρ* ≠ 0, it implies that at least one confounding variable exists that biases the ACME estimate. Larger values of *ρ*, in absolute value terms, imply larger bias in the ACME estimate. In the results section, we perform a sensitivity check by relaxing the assumption that *ρ* = 0 and re-estimate the system defined by equations (6) and (7) under varying values of *ρ*. Those estimates are consistent only if one assumes that the value of *ρ* used in the model is the true correlation.

## Results

In this section, we first present descriptive results about changes in outcomes by treatment status, and then present impact estimates of the REU on nutritional knowledge indicators and adoption behavior. We next use causal mediation analysis to ascertain how much of the adoption behavior can be explained through the knowledge of messages regarding the health benefits of vitamin A, and what role both adoption and nutrition knowledge play in explaining improved vitamin A intakes. We also describe sensitivity analyses both for attrition and for the sequential ignorability assumption.

### Descriptive Results

First, in panel A of table 2 we compare baseline and endline values for all three classes of outcome variables by treatment group. Descriptively, we find substantial evidence of impacts in both countries. First, the REU also appears to have affected adoption in both countries. In Mozambique, 75% and 79% of farmers in IT and MT were growing OFSP at endline, whereas only 9% of farmers in the control group were doing so. In Uganda, adoption rates at endline were 66% and 62% in IT and MT, respectively, and 6% in the control group. Among farmers growing OFSP, the share of OFSP in sweet potato cultivated area is between 70% and 73% at endline, whereas it is 7% among the control group. In Uganda, the share is between 44% and 47% at endline compared with 2% among the control group. Moreover, these figures mask substantial differences in distributions; whereas a large share of sweet potato farmers only grow OFSP at endline in Mozambique, many farmers in Uganda split their fields between OFSP and other SP.

**Table 2:** Average Baseline and Endline Outcomes, by Treatment Group, REU, Mozambique and Uganda

Outcome	Mozambique			Uganda		
	IT	MT	Control	IT	MT	Control
**Panel A: Adoption Indicators**						
Growing OFSP						
Endline	0.75	0.79	0.09	0.67	0.63	0.05
Share of OFSP in sweet potato area						
Baseline	0.19	0.11	0.12	0.00	0.00	0.005
Endline	0.73	0.70	0.07	0.46	0.44	0.02
Total reported area, OFSP (acres)						
Baseline	0.072 (0.320)	0.046 (0.280)	0.021 (0.148)	0.00 (0.00)	0.00 (0.00)	0.0005 (0.008)
Endline	0.114 (0.209)	0.118 (0.253)	0.017 (0.114)	0.176 (0.262)	0.121 (0.156)	0.008 (0.057)
**Panel B: Nutritional Knowledge Indicators**						
Knows OFSP has vitamin A						
Baseline	0.11	0.21	0.17	0.09	0.12	0.06
Endline	0.68	0.63	0.36	0.72	0.71	0.26
Number of Vitamin A Facts Known						
Baseline	0.71 (0.63)	0.73 (0.60)	0.73 (0.62)	0.94 (0.68)	0.96 (0.71)	0.94 (0.68)
Endline	1.28 (0.68)	1.47 (0.76)	0.91 (0.66)	1.46 (0.79)	1.51 (0.78)	0.91 (0.70)
**Panel C: Dietary Intakes, Reference Children**						
Vitamin A (mcg RAE)						
Baseline	210.6 (195.2)	202.5 (223.0)	187.1 (188.0)	528.5 (930.2)	409.7 (427.8)	563.9 (1177.2)
Endline	646.4 (838.6)	629.3 (731.4)	353.4 (613.7)	885.3 (1142.7)	1096.8 (1603.5)	595.5 (815.9)

*Note:* For continuous outcomes, standard deviations appear in parentheses. Share of OFSP in sweet potato area is conditional on growing OFSP. Reference children were aged 6–35 months at baseline in Mozambique and 36–71 months at baseline in Uganda. In Mozambique, sample size is 628 for the adoption indicators, 610 for the nutrition knowledge indicators, and 379 for the dietary intakes. In Uganda, sample size is 975 for the adoption and nutrition indicators, and 446 for the dietary intakes.

*Source:* REU Baseline and Endline Survey Data, Mozambique and Uganda.

Table 2, panel B shows that nutritional knowledge also improved in both countries. In Mozambique, approximately two-thirds of mothers in the two treatment groups name OFSP as a source of vitamin A at endline, whereas only one-third of mothers in the control group do so. Less than 20% of mothers did so prior to baseline. The pattern of learning was similar in Uganda. We find similar improvements in the number of vitamin A messages that women can recite.

Finally, in panel C we show that among reference children, average dietary intakes of vitamin A increased substantially in the two treatment groups in both countries relative to the control group. At baseline, reference children consumed approximately 200 *μg* retinol activity equivalents (RAE) of vitamin A in Mozambique, and between 430 and 550 *μg* RAE per day in Uganda; reference children in Uganda were older and therefore had higher consumption. At endline, reference children in both countries assigned to IT and MT consumed significantly more vitamin A than children in the control groups.

### Primary REU Impact Estimates

Estimating equation (1) with an indicator for adoption as the dependent variable demonstrates that IT and MT has remarkably similar impacts on OFSP adoption in both countries. Results in panel A of table 3 show that in Mozambique, when additional household characteristics are not included, households in IT and MT were 65.7 and 69.2 percentage points more likely to adopt than the control group, respectively. When additional covariates are included, these estimates are slightly lower.^14^ In Uganda, households in IT and MT are 61.7 and 57.9 percentage points more likely to adopt OFSP (columns 7 and 8) than the control group, when we do not control for additional household characteristics. When we do so, the coefficients on the treatment indicators slightly increase. Therefore in both countries, the REU was successful in leading to OFSP adoption among farmers. Furthermore, as point estimates for adoption were similar in both countries (last row of panel A), we can combine the two treatment groups for causal mediation analysis. The combined impact estimates using equation (2) and shown in panel B are 63.9 percentage points in Mozambique and 61.7 percentage points in Uganda (columns 2 and 8, respectively).

**Table 3:** Impacts of REU Intensive and Moderate Treatments on Adoption Measures at Endline, Mozambique and Uganda

	Mozambique						Uganda					
	Adopted OFSP		Share of OFSP in SP Area		Total Area, OFSP (acres)		Adopted OFSP		Share of OFSP in SP Area		Total Area, OFSP (acres)	
	(1)	(2)	(3)	(4)	(5)	(6)	(7)	(8)	(9)	(10)	(11)	(12)
**Panel A: Intensive Treatment versus Moderate Treatment**												
Intensive Treatment	0.659*** (0.043)	0.629*** (0.046)	0.647*** (0.040)	0.622*** (0.042)	0.091*** (0.017)	0.095*** (0.015)	0.617*** (0.040)	0.624*** (0.030)	0.438*** (0.027)	0.428*** (0.023)	0.168*** (0.018)	0.169*** (0.017)
Moderate Treatment	0.691*** (0.033)	0.650*** (0.037)	0.612*** (0.033)	0.587*** (0.033)	0.100*** (0.021)	0.092*** (0.020)	0.579*** (0.071)	0.595*** (0.039)	0.414*** (0.039)	0.410*** (0.040)	0.113*** (0.015)	0.117*** (0.014)
Additional Covariates?	No	Yes	No	Yes	No	Yes	No	Yes	No	Yes	No	Yes
Test H_0_: Model 1 = Model 2 (p-value)	0.464	0.625	0.438	0.449	0.731	0.883	0.649	0.542	0.615	0.688	0.016	0.018
**Panel B: Average treatment effect of both interventions**												
Treated	0.676*** (0.032)	0.639*** (0.036)	0.628*** (0.029)	0.604*** (0.030)	0.096*** (0.015)	0.094*** (0.015)	0.607*** (0.034)	0.617*** (0.025)	0.432*** (0.023)	0.424*** (0.020)	0.154*** (0.014)	0.156*** (0.014)
Additional Covariates?	No	Yes	No	Yes	No	Yes	No	Yes	No	Yes	No	Yes
Endline Control Mean	0.093		0.073		0.017		0.054		0.027		0.008	
Number of obs.	610	610	534	534	610	610	975	975	751	751	975	975

*Note:* All models are single difference models at endline. Baseline levels of adoption and area planted with OSP were very low, and so were omitted from these models. The share of OFSP in SP area has 59 missing observations in Mozambique and 224 missing observations in Uganda because these households did not grow any sweet potato. Tests of equality of impact of Model 1 and Model 2 are adjusted Wald tests. Average treatment effects reported at the bottom of the table are average impacts over Model 1 and Model 2 using the same specification for that column in a separate regression. Additional covariates included in some specifications, all measured at baseline, are whether the household had access to off-farm work, the number of male and female adults, whether the household head was male, whether or not a nutrition promoter lived in the household, whether the household grew sweet potato in 2006, and per-capita expenditures. All regressions include strata-level fixed effects. Standard errors are clustered at the village level in Mozambique and the farmer group level in Uganda. Asterisks ***indicate significance at the 1% level. For Mozambique, wild bootstrapped p-values appear in appendix table S.1.

*Source:* REU Baseline and Endline Survey Data, Mozambique and Uganda.

Next, we estimate the intensity of impact of IT and MT using the share of OFSP in SP area and the total cultivated area of OFSP.^15^ As shown in column 4 of table 3, IT and MT farmers in Mozambique devote 62.2 and 58.7 percentage points more of their sweet potato area to OFSP, respectively. About half the sample in Mozambique grew sweet potatoes prior to baseline, so it is not surprising that the coefficient is relatively large. Many farmers adopted OFSP as their only sweet potato between baseline and endline; about half of IT and MT farmers exclusively grew OFSP at endline. So some substitution from other SP took place as well as some sown area expansion, in lieu of substituting for other, potentially more nutritious crops. In Uganda, farmers were more likely to grow sweet potatoes prior to the baseline, so not surprisingly the share of sweet potato area devoted to OFSP only rises by between 41.0 and 42.8 percentage points among IT and MT relative to the control group, respectively (column 10). In Uganda, many households demonstrated a preference for variety, keeping more than half of their sweet potato fields in conventional varieties. Results are qualitatively similar in both countries when intensity is measured using the total area under OFSP in table 3 (columns 5, 6, 11, and 12). In general, there are again no significant differences between models, so as with the discrete adoption indicator, the two estimates can be combined into one treatment indicator without loss of generality (panel B).

Using equations (1) and (2), in table 4 we next estimate impacts for the two nutrition knowledge indicators. In Mozambique, for both specifications, the estimates show that the REU had a significant impact on the proportion of mothers who named OFSP as a source of vitamin A, as well as on the number of vitamin A facts known. In Uganda, point estimates are also statistically significant for both dependent variables and somewhat higher than in Mozambique.^16^ In Uganda, the proportion of mothers naming OFSP as a source of vitamin A increased by about 45 percentage points in both IT and MT, and the number of messages known also increased by over half a message, on average. In neither country do we find larger point estimates for IT than MT; in fact, differences in coefficients between models are either not statistically significant or coefficients are actually higher among MT households. Using equation (2), the proportion of mothers naming OFSP as a source of vitamin A increased by 24.4 and 45.4 percentage points in Mozambique and Uganda, respectively (columns 2 and 6 of panel B), similarly, mothers knew 0.35 and 0.57 more vitamin A facts as a result of the program in Mozambique and Uganda (column 4 and 8 of panel B). Therefore, there are some clear, if modest, gains in nutritional knowledge that occurred among mothers during the REU in both countries. However, these changes are not important if they do not lead to changes in behavior, whether they affect production or consumption of OFSP.

**Table 4:** Impacts of REU Intensive and Moderate Treatment Models on Nutritional Knowledge Indicators at Endline, Mozambique and Uganda

Variable	Mozambique				Uganda			
	Knows OFSP a source of vitamin A, 2009		Number of vitamin A Facts Known, 2009		Knows OFSP a source of vitamin A, 2009		Number of vitamin A Facts Known, 2009	
	(1)	(2)	(3)	(4)	(5)	(6)	(7)	(8)
**Panel A: IT versus MT**								
Intensive Treatment	0.328*** (0.047)	0.281*** (0.051)	0.370*** (0.091)	0.257*** (0.086)	0.456*** (0.040)	0.457*** (0.030)	0.554*** (0.063)	0.559*** (0.062)
Moderate Treatment	0.266*** (0.052)	0.206*** (0.054)	0.558*** (0.087)	0.435*** (0.086)	0.441*** (0.059)	0.447*** (0.039)	0.603*** (0.114)	0.613*** (0.106)
Additional Covariates?	No	Yes	No	Yes	No	Yes	No	Yes
Test H_0_: Model 1 = Model 2(p-value)	0.299	0.184	0.026	0.017	0.820	0.811	0.690	0.633
**Panel B: Average treatment effect of both interventions**								
Treated	0.296*** (0.041)	0.243*** (0.045)	0.469*** (0.082)	0.347*** (0.081)	0.452*** (0.036)	0.454*** (0.028)	0.566*** (0.058)	0.573*** (0.057)
Additional Covariates?	No	Yes	No	Yes	No	Yes	No	Yes
Endline Control Mean	0.358		0.913		0.258		0.856	
Number of obs.	610	610	610	609	975	975	975	975

*Note*: Regressions are ANCOVA models controlling for baseline level of the outcome. Tests of equality of impact of Model 1 and Model 2 are adjusted Wald tests. Average treatment effects reported at the bottom of the table are average impacts over Model 1 and Model 2, using the same specification for that column in a separate regression. Additional covariates are listed in the notes for table 4. All regressions include strata-level fixed effects. Standard errors are clustered at the village level in Mozambique and the farmer group level in Uganda. Asterisks ***, **, and * indicate significance at the 1%, 5%, and 10% levels, respectively.

*Source:* REU Baseline and Endline Survey Data, Mozambique and Uganda.

Hotz et al. (2012a,b) documents the substantial increase in average dietary intakes of vitamin A for reference children in Mozambique and Uganda, respectively.^17^ In neither country do we find a statistically significant difference in impact magnitudes across IT and MT, suggesting that the more intensive and expensive intervention in IT did not contribute to additional improvements in vitamin A intakes. In Mozambique the average impact is 217.9 *μg* RAE per day for children aged 6–35 months at baseline, or almost exactly the US recommended daily intake for this age group (210 *μg* RAE).^18^ In Uganda, the average impact on dietary intakes of vitamin A for children 36–71 months at baseline is somewhat larger at 390.7 *μg* RAE, which is much higher than the average requirements for this age group. The larger effect in Uganda in part reflects the fact that the reference children are older, and so consume more food in general.

There are two important implications. First, for causal mediation analysis it should not matter that we average impacts among IT and MT. Second, MT was explicitly designed to be less costly than IT, implying that MT was more cost effective than IT. We therefore only consider the costs of the MT model when considering cost effectiveness.

### Attrition

A standard concern is whether attrition affects basic treatment impacts, as about 10% and 8% of the baseline sample attrited in Mozambique and Uganda, respectively. We initially test whether attrition is random by estimating a probit model using an indicator variable for staying in the sample by endline as the dependent variable, and indicator variables for the two treatments and a consistent set of covariates as explanatory variables.^19^ Whereas we do not find a relationship in table 5 between treatment status and attrition in Mozambique (columns 1–2), in Uganda we find that either treatment status is negatively associated with attrition, holding other things constant (columns 3-4), indicating that in Uganda, attrition could influence findings. Consequently, we estimate Lee (2009) bounds for the main coefficient estimates on both discrete adoption and nutrition knowledge variables, which can be found in table S.2 of the supplementary online appendix.^20^ For all three variables and both countries, 95% confidence intervals do not include zero. For Uganda, we also re-estimated impacts on all adoption and nutritional knowledge variables using inverse probability weighting to adjust results for nonrandom attrition; results shown in the on-line appendix are quite similar to those discussed above.

**Table 5:** Attrition and Sample Size, Mozambique and Uganda

Variable	Mozambique		Uganda	
	(1)	(2)	(3)	(4)
Intensive Treatment	−0.024 (0.029)	−0.024 (0.030)	−0.081*** (0.019)	−0.081*** (0.019)
Moderate Treatment	0.015 (0.034)	0.015 (0.032)	−0.034*** (0.010)	−0.034*** (0.010)
Number of Male Adults	0.013 (0.010)	0.014 (0.009)	−0.001 (0.004)	−0.001 (0.004)
Number of Female Adults	−0.001 (0.009)	0.0002 (0.010)	−0.018** (0.006)	−0.018** (0.006)
Female Head?	−0.119** (0.064)	−0.116** (0.065)	0.007 (0.014)	0.007 (0.014)
Log, monthly per capita expenditures	0.010 (0.014)	0.011 (0.014)	0.011* (0.006)	0.010* (0.006)
Access to Off-farm work	−0.035 (0.024)	−0.028 (0.023)	−0.006 (0.007)	−0.006 (0.007)
Grew Sweet Potato at baseline?^a^	−0.003 (0.019)	−0.008 (0.018)	0.001 (0.015)	0.001 (0.015)
Access to lowlands?	0.051** (0.023)	0.052** (0.022)	0.006 (0.010)	0.006 (0.009)
Number of vitamin A messages known		−0.046** (0.019)		0.003 (0.006)
Number of obs., baseline	703	703	1048	1048
Number of obs., endline	628	628	975	975

*Note:* Standard errors clustered at the village level in Mozambique and the farmer group level in Uganda. Superscript ^a^ indicates that growing sweet potato at baseline is represented by area under sweetpotato at baseline. Asterisks * and ** indicate significance at the 10% and 5% levels, respectively. Estimation by probit model and marginal effects are presented. Strata-level dummy variables are also included in both specifications.

*Source:* REU Baseline and Endline Survey Data, Mozambique and Uganda.

### Causal Mediation Analysis: Estimates

To understand the contributions of additional nutritional knowledge to the adoption decision, we make the sequential ignorability assumption embedded in equation (5). After estimating equations (6) and (7), we provide conditional correlations between the error terms of the two equations to understand whether bias might exist in our estimates of the ACME, and if so, in which direction the bias might be.

The sequential ignorability assumption is implausibly strong, so it is important to think through how unobservables would affect estimates. We provide a sensitivity analysis of the ACME estimate as follows. After estimating the base model, we relax the assumption that *ρ* = 0. We then specify hypothetical values for *ρ* and re-estimate the ACME from equations (6) and (7) assuming that correlation, with bootstrapped 95% confidence intervals. We graph each of these estimates for both adoption and vitamin A intakes as outcomes, with the number of vitamin A messages recalled as the mediator. These graphs show how sensitive the ACME estimates are to potential correlation in the residuals.

We initially estimate causal mediation effects making the linearity assumption on the discrete adoption measure, using both nutritional knowledge variables as mediators. Coefficient estimates shown in table 6 suggest a very limited amount of adoption occurs through nutritional knowledge, irrespective of the mediating variable.^21^ We find a positive but insignificant coefficient (0.058) on the OFSP as a vitamin A source mediating variable in Mozambique (panel A, column 2), and a statistically significant coefficient in Uganda of 0.098 (panel A, column 6). In Mozambique, controlling for baseline characteristics the point estimate for the effect of the number of vitamin A messages known at endline on OFSP adoption is 0.049 (column 4); in Uganda, it is 0.040 (column 8). In panel B, we calculate the ACME and ADE for both mediating variables. Whether or not we condition on baseline characteristics, the mediating effect of the nutrition variables never exceeds 5% of the average total effect in Mozambique (in column 3) and 12% in Uganda (in column 5). As mediating variables, increased knowledge appears to have had only limited importance for OFSP adoption in both countries.

**Table 6:** Average Impacts of REU on Adoption at Endline, Including Nutrition Knowledge Mediating Variables, and Estimates of ACME and ADE for the Role of Nutrition Knowledge in OFSP Adoption, Mozambique and Uganda

	Mozambique				Uganda			
	(1)	(2)	(3)	(4)	(5)	(6)	(7)	(8)
**Panel A: Coefficient Estimates**								
Treated	0.654*** (0.037)	0.624*** (0.040)	0.640*** (0.037)	0.622*** (0.038)	0.531*** (0.037)	0.572*** (0.029)	0.576*** (0.036)	0.594*** (0.027)
Knows OFSP is source of vitamin A, endline	0.073* (0.039)	0.058 (0.041)			0.166*** (0.032)	0.098*** (0.032)		
Number of vitamin A facts known, endline			0.074*** (0.023)	0.049** (0.022)			0.056*** (0.017)	0.040** (0.015)
Number of Obs.	610	610	610	609	975	975	975	975
R^2^	0.419	0.447	0.428	0.450	0.399	0.461	0.383	0.457
**Panel B: Estimate of ACME and ADE**								
Treatment effect on knowledge	0.296	0.243	0.469	0.347	0.452	0.454	0.567	0.573
ACME	0.022** (0.011)	0.014 (0.009)	0.035** (0.013)	0.017* (0.009)	0.075*** (0.016)	0.044*** (0.015)	0.031*** (0.010)	0.023** (0.009)
ADE	0.654*** (0.041)	0.625*** (0.040)	0.641*** (0.036)	0.622** (0.038)	0.532*** (0.037)	0.563*** (0.036)	0.576*** (0.036)	0.584*** (0.035)
Share of Treatment Effect, Vitamin A Messages	3.3	2.1	5.3	2.6	12.3	7.2	5.1	3.7
Additional Covariates?	No	Yes	No	Yes	No	Yes	No	Yes
Correlation, residuals	<0.0001	<0.0001	<0.0001	<0.0001	−0.0019	0.0007	0.0027	−0.0005

***Note:*** Standard errors are clustered at the village level in Mozambique and the farmer group level in Uganda. Additional covariates are listed in the notes for table 4; treatment effects on knowledge are from panel B of table 4. All regressions include strata-level fixed effects and lagged values of the mediating variable. Asterisks ***, **, and * indicate significance at the 1%, 5%, and 10% levels.

***Source:*** REU Baseline and Endline Survey Data, Mozambique and Uganda.

**Figure 1:**
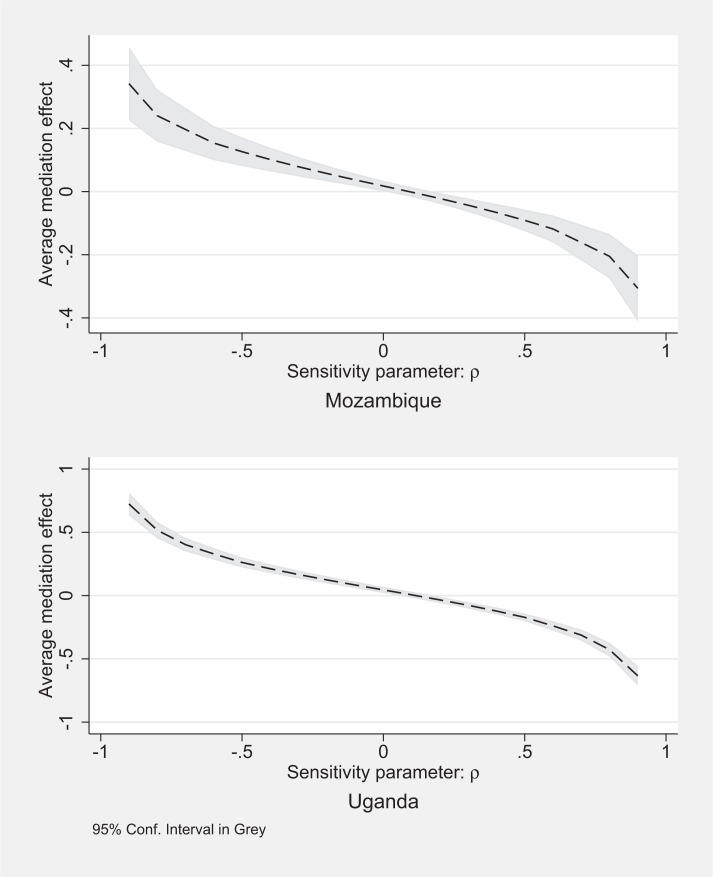
Sensitivity Analysis, using number of vitamin A messages as mediator variable and adoption as the outcome area, including interaction terms, Mozambique and Uganda

When we relax the sequential ignorability assumption and assume varying correlation of the error terms in equations (6) and (7), in figure 1 we find that the correlation between residuals have to be large and negative for the mediating variable to have a strong impact on adoption in both countries.^22^ In Mozambique, for example, even a correlation of *ρ* = −0.5 would not explain a large amount of adoption (figure 1, top half). In both countries, if *ρ* is positive, then the ACME is actually overestimated.

Therefore, it is worth discussing the most plausible direction of correlation between the error terms. Recall the REU provided households with both OFSP vines and nutritional knowledge. The residuals in explaining nutritional knowledge, then, are the amount of increased nutritional knowledge that cannot be explained after controlling for the treatment effect and baseline household characteristics, and the residuals in explaining adoption are the amount of adoption that cannot be explained after controlling for the same variables and the mediating variable. If anything, these residuals should be positively correlated since a negative correlation would imply that households with additional unexplained nutritional knowledge are actually less likely to have unexplained adoption behavior. One would expect that if any residual correlation exists it would be positive, as households likely to participate in one part of the intervention for unobserved reasons would also be likely to participate in the other part.

In fact, we estimated correlations between residuals for the regressions explaining mediating variable and residuals from the equations estimated in table 6, and find small positive correlations in Uganda, and no correlations at all in Mozambique. These conditional correlations suggest that if anything, we overestimate the ACME in Uganda, but not in Mozambique. In Uganda, the correlations are slightly higher for the variable measuring knowledge that OFSP is a source of vitamin A, suggesting that if we want to conclude that 12% of adoption in Uganda can be explained through increased knowledge, it should be considered an upper bound.

In summary, using the available measures we find that the demand creation, at least via its effect on knowledge about the nutritional benefits of OFSP, had little impact on adoption. It could be that other aspects of the project, such as the initial price of vines (zero), the vines’ other traits such as resistance to pests, or consumer preference for OFSP were simply important enough to catalyze strong OFSP adoption. Further, it could be that enhancing nutritional knowledge was not necessary for project success. An alternative explanation is that variables representing project messages may not be broad enough to reflect project impacts through increased knowledge; for example, the general message that OFSP is healthy might have been an important driver of adoption. That message, however, is not simple to measure quantitatively given the available data. We return to this point as we discuss the cost effectiveness implications of the results.

Next, we strive to understand the role of both adoption and nutritional knowledge in explaining vitamin A intakes among reference children in both Mozambique and Uganda. We initially estimate a version of equation (7) with two potential mediating variables, one measuring adoption and one measuring nutritional knowledge.^23^ We build up estimates in both countries by first estimating models with each mediating variable alone, then testing each possible nutritional knowledge indicator. We use the discrete measure of adoption, and test both possible measures of nutritional knowledge; given that we are primarily using binary mediating variables, we continue to make both the linearity and sequential ignorability assumptions, so results remain conditional on those assumptions.

**Figure 2:**
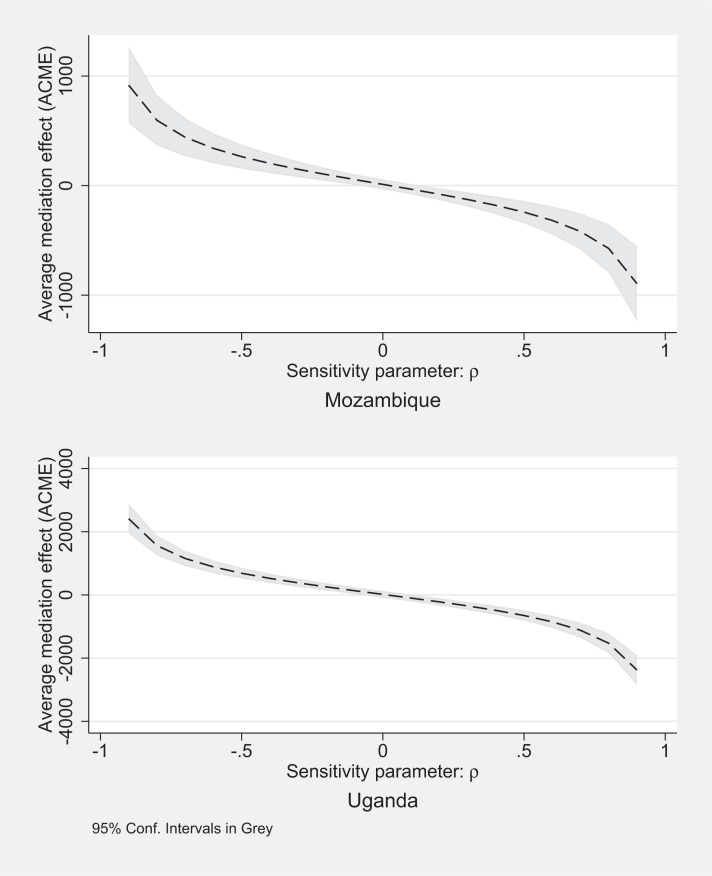
Sensitivity Analysis, using number of vitamin A messages as mediator variable, for vitamin A intakes among reference children as the outcome variable, including interaction terms, Mozambique and Uganda

In Mozambique, when we add the adoption variable as a mediator in a regression explaining vitamin A intakes, in table 7 we find that it explains nearly the entire treatment effect (column 1). When we instead use one of the two nutrition knowledge indicators as a mediating variable (columns 2 and 3), the coefficient estimates on the mediating variables are relatively small and imprecisely estimated, both with *t* ratios below 1. As with adoption, the sensitivity analysis using the number of vitamin A facts known as the mediator in figure 2 demonstrates that there would again have to be a relatively strong, negative correlation between error terms to generate a large mediation effect through nutritional knowledge (top half). Therefore, it seems like the demand creation component had little to do with increasing vitamin A intakes.

**Table 7:** Estimates of ACME and ADE for the Role of Adoption and Nutrition Knowledge in Vitamin A Intakes among Reference Children, REU, Mozambique and Uganda

	Mozambique					Uganda				
	(1)	(2)	(3)	(4)	(5)	(6)	(7)	(8)	(9)	(10)
**Panel A: Coefficient Estimates**										
Treated	36.9 (100.1)	207.0** (81.1)	205.3** (81.8)	32.0 (104.1)	35.8 (102.0)	193.5* (114.0)	377 9*** (104.5)	321 4*** (102.1)	186.1 (116.1)	141.2 (121.7)
Plans to conserve vines or planted OFSP this season	272.3*** (90.4)			270.1*** (89.4)	270.5*** (91.0)	324.0** (129.8)			323.0** (130.5)	305.0** (124.5)
Knows OFSP is source of vitamin A, endline		43.0 (71.1)		24.9 (67.3)			31.3 (116.7)		18.0 (117.9)	
Number of vitamin A facts known, endline			36.3 (70.0)		6.5 (72.4)			129.7 (89.0)		117.6 (88.6)
**Panel B: Estimates of ACME and ADE**										
ACME, Adoption	180.6*** (60.3)			179 2*** (59:5)	179.4*** (60.5)	198.6** (78.5)			197.9** (78.9)	186.92** (75.5)
ACME, Vitamin A Messages		10.9 (18.4)	12.4 (24.9)	6.31 (17,4)	2.32 (25.8)		14.1 (52.5)	70.8 (52.6)	8.09 (52.9)	64.23 (54.1)
ADE	37.3 (100.0)	207.0** (80.9)	205.5** (81.6)	32.4 (104.0)	36.2 (102.0)	193.3* (114.2)	377.8*** (104.5)	3211*** (102.2)	185.9 (116.3)	140.74 (121.9)
Share of Treatment Effect, Adoption	82.9			82.2	82.3	50.7			50.5	47.7
Share of Treatment Effect, Vitamin A Messages		5.0	5.7	2.9	1.1		3.6	18.1	2.1	16.4
Additional Covariates in all regressions?	yes	yes	yes	yes	yes	yes	yes	yes	yes	yes
Number of observations	372	372	372	372	372	446	446	446	446	446

*Note:* Models in both countries include district (strata) dummy variables. Standard errors are clustered at the village level in Mozambique and the farmer group level in Uganda, and are generated on the ACME and ADE using seemingly unrelated regressions. Regressions underlying the mediation effects include baseline value of vitamin A intakes and additional covariates included in all regressions. Asterisks ***,**, and * indicate significance at the 1%, 5%, and 10% levels, respectively.

*Source:* REU Baseline and Endline Survey Data, Mozambique and Uganda.

To confirm this hypothesis, we use the discrete adoption variable and the two nutrition knowledge variables sequentially as multiple mediators. The coefficient estimate on the adoption variable shown in table 7 is nearly the same in both specifications as when it was used alone, and the estimated coefficients on the nutrition knowledge variables remain relatively small and are not statistically different from zero (columns 4 and 5). The corresponding ACME for adoption suggests that about 82% of intakes by reference children can be explained through adoption, while only 1% to 3% can be directly explained through nutritional knowledge. These results appear quite consistent with adoption results, which suggested that nutritional knowledge only had a small impact on adoption, if any, nor was there much effect on vitamin A intakes among reference children.

In Uganda (columns 6 through 10 of table 7), as in Mozambique, we find a large, statistically significant coefficient estimate on the adoption variable (column 6). However, the point estimate on the treatment effect remains reasonably large, suggesting unexplained variation in vitamin A intakes. Using the nutritional knowledge variables as mediators (columns 7 and 8), point estimates for both coefficients are not statistically different from zero. Graphing the average causal mediation effect and the average direct effect in figure 2, not surprisingly we find that the number of vitamin A facts known does not appear to be a mediator. Similar to Mozambique, the error terms would have to have a strong negative correlation before the mediation effect through nutritional knowledge would explain a large amount of the average treatment effect for dietary intakes of vitamin A.

When we estimate models with two mediating variables in Uganda, coefficient estimates on the mediating variables remain similar to regressions in which they entered alone. In table 7, we show that the ACME for adoption explains 43% to 47% of the treatment effect on vitamin A intakes (columns 9 and 10), whereas vitamin A messages explain 2% or 16% of the treatment effect, depending upon the mediator; note that neither coefficient estimate is statistically different from zero, so these estimates are not precise. The ADE, then, remains quite large in both specifications. Relative to Mozambique, there is a reasonably large amount of the treatment effect that remains unexplained by the two mediating variables. Since the direct pathway from the program to vitamin A intakes is unlikely to be large, these results suggest some variable is missing that might help explain intakes by reference children.^24^ A reasonable hypothesis is that an unobservable related to demand creation, such as the general message that OFSP are healthy, might explain the ADE. It is not likely that other creation messages disseminated by the project explain the ADE, such as messages about young child feeding practices or hygiene, as no treatment effects were found for such behavior change (de Brauw et al. 2010).

## Cost Effectiveness Implications

To examine the implications of our results for the cost effectiveness of future, similar interventions, we primarily focus on the average costs per beneficiary since the marginal costs are difficult to define in this context (see de Brauw et al. 2010, for an extended discussion). We measure costs per program beneficiary rather than attempting to estimate aggregate public and private benefits because both are difficult to define and measure, and would require a large set of assumptions. We only consider costs for MT since it was less expensive and both models had similar impacts.

The REU program beneficiaries are measured in four different ways. First, direct beneficiaries are households that received vines from the project. In both countries, organization or farmer group membership was somewhat fluid, so we use aggregates from initial project vine distribution lists to estimate the number of direct beneficiaries across the two models. Second, other households also benefited from the project through vines given to them by direct beneficiaries.^25^ In both countries, we measured such indirect beneficiaries: among MT households, in Mozambique, 0.32 additional households obtained vines per beneficiary household, whereas one additional household obtained vines in Uganda (see appendix table A.2).

The third and fourth definitions of beneficiaries are individuals since the target beneficiaries of the REU are mothers and children for whom increased vitamin A consumption is most important; we estimate their number in the intervention households. Ugandan households are somewhat larger than Zambézian households; in Uganda there are 1.73 children aged under 5 per household, whereas in Mozambique there are 1.25 children aged under 5. Based on the average of 0.97 mothers per household in Mozambique and 0.99 in Uganda, we assume there are approximately 2.22 beneficiaries per household in Mozambique and 2.69 beneficiaries per household in Uganda. Since not all beneficiaries actually adopt OFSP, we also estimate the benefits per adopting household based on single difference estimates of adoption impacts.

On a per household or per beneficiary basis, shown in panel A of table 8, MT was slightly more expensive in Mozambique than in Uganda ($146 versus $132 per household). On per individual beneficiary basis and accounting for diffusion, costs drop to $52 in Mozambique and $25 in Uganda. The intervention appears less expensive, in relative terms, in Uganda as the number of direct beneficiaries per household were higher, as was diffusion. Clearly, increasing diffusion can help make the costs per beneficiary lower. Once we account for the fact that not all households that benefit from the project actually adopt vines in panel B, the cost per individual beneficiary increases to $68 in Mozambique and $36 in Uganda. About 70% of the disparity between countries is due to the difference in diffusion rates.

**Table 8:** Average Costs per Beneficiary Household and Individual, REU and Hypothetical Reduced REU Programs, all Based on MT, Mozambique and Uganda

Average Costs per:	Mozambique	Uganda
*Panel A: Targeted Household*		
Direct Household Beneficiary	$146	$132
Direct Individual Beneficiary	$65	$49
Direct+Indirect Household Beneficiary	$117	$66
Direct+Indirect Individual Beneficiary	$52	$25
*Panel B: Households Adopting OFSP*		
Direct Household Beneficiary	$191	$199
Direct Individual Beneficiary	$85	$74
Direct+Indirect Household Beneficiary	$153	$100
Direct+Indirect Individual Beneficiary	$68	$36
*Panel C: Households Adopting OFSP*		
Direct Household Beneficiary	$170	$157
Direct Household Beneficiary, dropping 25 percent of demand creation	$156	$145
Direct Household Beneficiary, dropping 50 percent of demand creation	$141	$132
Direct Household Beneficiary, dropping 75 percent of demand creation	$127	$120

*Note*: Panel A gives costs per targeted household, meaning all households in areas included in the hypothetical intervention. Panel B gives costs per adopting household, which assumes the adoption rate in Model 2 of the REU. Panel C hypothetically reduces costs of the actual REU by dropping components that do not appear to substantially affect adoption or vitamin A intakes.

**Figure 3:**
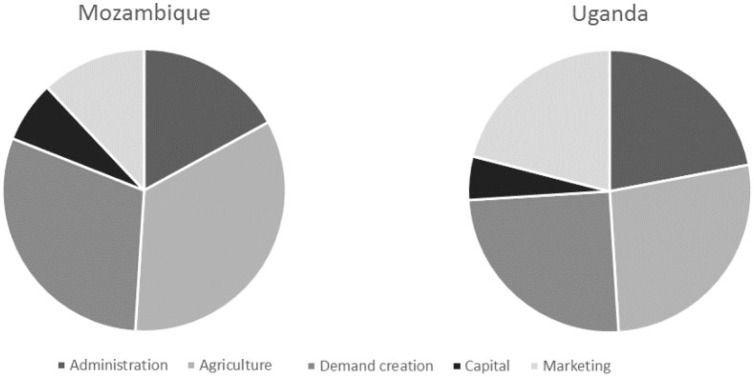
Budget shares of REU project components, Mozambique and Uganda

That being said, results also suggest that the implementation design could be modified without materially affecting impacts on adoption or dietary intakes. First, a reduced cost intervention could solely focus on OFSP production and consumption, rather than including a marketing component. Figure 3 shows REU budget proportions of each component; dropping the marketing component would reduce the budget by 11% in Mozambique and 21% in Uganda. In Mozambique, the results suggest the demand creation messages, mediated through nutrition knowledge, had minimal effects on both adoption and dietary intakes. In Uganda, there was more unexplained variation in vitamin A intakes; one hypothesis is that unmeasured, broader health messages of the project might have affected dietary intakes. So demand creation expenditures could be substantially scaled back in both countries, though one would caution that broader messages that OFSP are healthier than white or yellow sweet potatoes should remain part of the program, as we cannot say much about their effectiveness.

We consider the implications of removing the marketing component and cutting the demand creation budget by 25%, 50%, and 75%. The latter two amounts would heavily cut back on the nutrition extension, but would retain basic messages and potentially mass marketing to promote project nutrition messages. Average costs per adopting household would drop to between $127 and $170 in Mozambique and $120 and $157 in Uganda, depending upon the budget reduction, as shown in panel C of table 8. A 50% reduction is probably the largest feasible while maintaining demand creation contact with households, so costs per adopting household could be reduced to $141 in Mozambique and $132 in Uganda. In both countries, an increased emphasis on promoting diffusion would help decrease average cost estimates even further.

## Conclusions

In this article, we quantify the impacts of an integrated biofortification program that delivers two models varying in intensity using a randomized control trial conducted in both Mozambique and Uganda. Results show that in both countries, despite quite different initial conditions, a substantial share of farmers adopted OFSP, mothers learned about the contribution of the crops to child health, and vitamin A intakes increased among target children. Equally important, the MT model worked just as well as the IT model. The results on OFSP adoption demonstrate that crop introductions can be highly successful when new crops yield as well as old ones, and when consumer preferences are accounted for in crop development. Moreover, substantial OFSP adoption in Mozambique occurred despite sweet potato being a secondary staple there, providing encouraging evidence that biofortification can succeed even in areas where the target crop is not a major staple. Although treatment effects for vitamin A intakes were larger in Uganda than in Mozambique, as reference children were older and consumed more food in Uganda, the increase in consumption met vitamin A requirements in both countries.

We used causal mediation analysis to shed light on the mechanisms by which the program worked. In both countries, knowledge of the project’s primary nutritional messages appears to have had little direct effect on OFSP adoption. Conditional on the validity of assumptions made to generate the mediation results, we find that adoption is likely due to a combination of factors; OFSP are not that agronomically different from white sweet potatoes, and as people liked to consume them, it was not difficult to convince producers to produce them for own consumption in most cases. Since some work is necessary to maintain the vines over time, a lingering question is whether farmers will be able to sustain them over longer time periods.

We then examine the role that adoption and increased nutritional knowledge play in explaining increased vitamin A intakes among young children. After making strong assumptions related to causal mediation, we find that in Mozambique the increase is almost entirely explained by increased adoption, with nutritional knowledge playing a limited role. In Uganda, adoption was also the largest driver of higher intakes, but there was also a relatively large amount that was not explained by either mediation variable. A plausible explanation is that broader project messages, related to the fact that OFSP are healthy to consume, played a role in catalyzing consumption by younger children.

Finally, we discuss the implications of our results for the cost effectiveness of OFSP or biofortification programs now being planned or implemented. The MT was clearly more cost-effective than IT, and we suggest ways that future projects might further streamline the cost structure by focusing demand creation messages. Costs could be further reduced, at least in the short term, if farmers could be more actively induced to share OFSP planting material with non-project members since the project would benefit more farmers. Future research may focus on designing mechanisms to induce farmers to share OFSP planting material with others.

## Footnotes

1 Provitamin A refers to specific carotenoids produced by plants that are converted to vitamin A by humans after ingestion. The most abundant provitamin A carotenoid in sweet potato is *β*-carotene. The HarvestPlus-supported OFSP varieties in both countries are dense sources of *β*-carotene, were bred locally using conventional methods, and have good agronomic properties.

2 Jones and de Brauw (2015) further show that the REU in Mozambique also reduced diarrhea prevalence among children aged under 35 months in a repeated cross-section, and that reduction is attributable to increased OFSP intake.

3 In Mozambique, sample size was based on vitamin A intakes. In Uganda, blood samples were collected for serum retinol concentration, a measure of vitamin A status, for IT households, and vitamin A intakes based on the dietary intake survey were computed for MT households (it was too expensive to undertake biochemical analyses for all three treatment arms). These outcomes then formed the basis of the sample size determination. Hotz et al. (2012b) address the REU impact on serum vitamin A status.

4 In Mozambique, the study design passed the Internal Review Board for the Ministry of Health. In Uganda, the study was approved by the ethical review boards of the Makerere University Medical School and the Uganda National Council of Science and Technology. Both studies also passed IFPRI’s internal review board.

5 Organizations in Nicoadala and Mopeia were selected from a single stratum (the “South”).

6 To implement the REU in Mozambique, farmer groups or community organizations had to be formed by project staff, often from church groups. Before the fieldwork occurred in all communities, staff informed village leaders about the survey and compiled a list of households that were members of the primary community organization that would participate in the intervention. From that list of households, 25 households with children under three years old were randomly selected from the list of community group, where five were meant as replacement households, in case the community lists did not always accurately indicate households with children under 36 months old.

7 We discuss whether attrition might affect impact measurements when discussing the results.

8 The survey also included five households per farmer group that were neighbors to learn how the OFSP vines spread beyond direct REU beneficiaries within communities at endline.

9 Given that the project distributed vines to farmers in the last year of the REU in Mozambique, we deemed whether farmers kept vines to be a better indicator of adoption since maintaining vines through the dry season requires commitment and effort. Follow-up fieldwork conducted by the International Potato Center (CIP) in 2010 indicated that this variable reliably estimated community-level adoption.

10 All adoption variables are measured at endline only since OFSP were not widely available in survey areas of both countries at baseline.

11 Since the OFSP varieties that were disseminated varied in their content of *β*-carotene, the average *β*-carotene content of OFSP varieties being grown in each project district was estimated by collecting field samples of each variety and analyzing them in labs for *β*-carotene content, then using project data to estimate the proportion of each variety being grown and their yields, thus deriving a district and country-specific weighted average of vitamin A content.

12 A technical treatment of the framework can be found in de Brauw et al. (2015).

13 We also measure a direct effect of the intervention on consumption which could occur either because the project affected production or consumption for reasons not explicitly modeled, or because the proxy variables used in estimation do not fully capture project effects.

14 For Mozambique, statistical inference could be affected by the relatively small number of clusters. We therefore estimate *p*-values for all dependent variables using a wild bootstrap, as suggested by Cameron, Gelbach, and Miller (2008). We can reject all null hypotheses at the 1% level or better for the adoption, nutrition knowledge, and vitamin A outcomes; *p*-value estimates can be found in the supplementary appendix online.

15 For the former variable, regressions are conditional on growing any sweet potato, as observations drop when no area is devoted to sweet potatoes.

16 For the Uganda data, due to missing values for a number of control variables, we lose an additional 126 observations. Among households lost due to missing values, other average characteristics are not systematically different between the whole sample and the sample used in the regression analysis.

17 Results are shown in appendix table A.1.

18 For recommended daily intakes, see Food and Nutrition Board and Institute of Medicine (2001).

19 For both countries, we run the attrition regression both with and without a baseline indicator for the number of vitamin A messages known since between the two knowledge indicators, this variable exhibits the most variation at baseline.

20 We do not estimate Lee bounds for the share of OFSP in sweet potato area since those estimates are conditional on growing sweet potato.

21 The finding does not change when the share of OFSP in sweet potato area is used as the adoption measure, as shown in the supplementary appendix online.

22 In both figures 1 and 2, the confidence interval is generated from a bootstrap estimating the equation with a specific value of *ρ* using 100 samples with replacement.

23 Given that in the previous subsection we found that nutritional knowledge only has a small impact, if any, on adoption, we ignore the possibility that the effect of adoption on intakes flows through increased nutritional knowledge.

24 The estimated ADE could in part reflect unmodeled correlations between the residuals in error terms between the two mediating variables and the outcome variable.

25 Estimates of indirect beneficiaries are based on vine diffusion modules included in both endline surveys. Therefore, we may underestimate indirect beneficiaries if there was a great deal of consumption of OFSP by households purchasing OFSP in markets or receiving OFSP from direct beneficiary households. However, given that most intervention households in both countries largely grew OFSP for home consumption, the magnitude of our underestimate is likely small.

## Appendix

**Appendix Table A.1:** Impacts of REU Intensive and Moderate Treatment Models on Vitamin A Intakes at Endline, Reference Children, Mozambique and Uganda

	Mozambique		Uganda	
	(1)	(2)	(3)	(4)
**Panel A: IT versus MT**				
Intensive Treatment	300.5*** (85.9)	232.1** (84.8)	304.2** (115.6)	313.0*** (101.4)
Moderate Treatment	242.9** (86.4)	205.8** (78.5)	525.4** (220.4)	520.6*** (149.1)
Child Characteristics?	Yes	Yes	Yes	Yes
Additional Covariates?	No	Yes	No	Yes
Test H_0_: Model 1 = Model 2 (p-value)	0.472	0.693	0.358	0.178
**Panel B: Average treatment effect of both interventions**				
Treated	268.5*** (79.9)	217 9*** (75.8)	389.8*** (115.9)	391.9*** (97.0)
Child Characteristics?	Yes	Yes	Yes	Yes
Additional Covariates?	No	Yes	No	Yes
Number of Obs.	376	376	446	446

*Note*: Tests of equality of impact of Model 1 and Model 2 are adjusted Wald tests. Average treatment effects reported in panel B of the table are average impacts over Model 1 and Model 2, using the same specification for that column in a separate regression. Standard errors are clustered at the village level in Mozambique and the farmer group level in Uganda. Asterisks ***, **, and * indicate significance at the 1%, 5%, and 10% levels, respectively.

*Source:* REU Baseline and Endline Survey Data, Mozambique and Uganda.

**Appendix Table A.2:** Parameters for Diffusion and Primary Beneficiaries per Household, REU, Model 2, Mozambique and Uganda

Parameter	Mozambique	Uganda
OSP Diffusion Rate	0.32	1.00
Targeted Beneficiaries		
Mothers per Household	0.97	0.99
Children aged 6–59 months per Household	1.25	1.73
Total Beneficiaries per Household	2.22	2.72

*Note:* Diffusion rate is measured as the number of individuals with whom the household reported sharing OSP vines; figures based only on Model 2 households in both countries. Targeted beneficiaries are the total number of mothers and children between the ages of 6–59 months living in each household.
