# Supplementary material for: Biofortification, Crop Adoption and Health Information: Impact Pathways in Mozambique and Uganda
Source: Am J Agric Econ. 2018 Mar 15;100(3):906–30. doi: 10.1093/ajae/aay005 (PMC7053385; doi:10.1093/ajae/aay005)
Supplement: Supplementary file 1 [file AJAE-2018-AJAE-AAY005-S1.pdf]

AJAE appendix for "Biofortification, crop adoption and health information: Impact pathways in Mozambique and Uganda"

Alan de Brauw, Patrick Eozenou, Daniel O. Gilligan, Christine Hotz, Neha Kumar, and J.V. Meenakshi

December 2017

Note: The material contained herein is supplementary to the article named in the title and published in the American Journal of Agricultural Economics (AJAE).

Supplementary Figures

Panel A. Mozambique

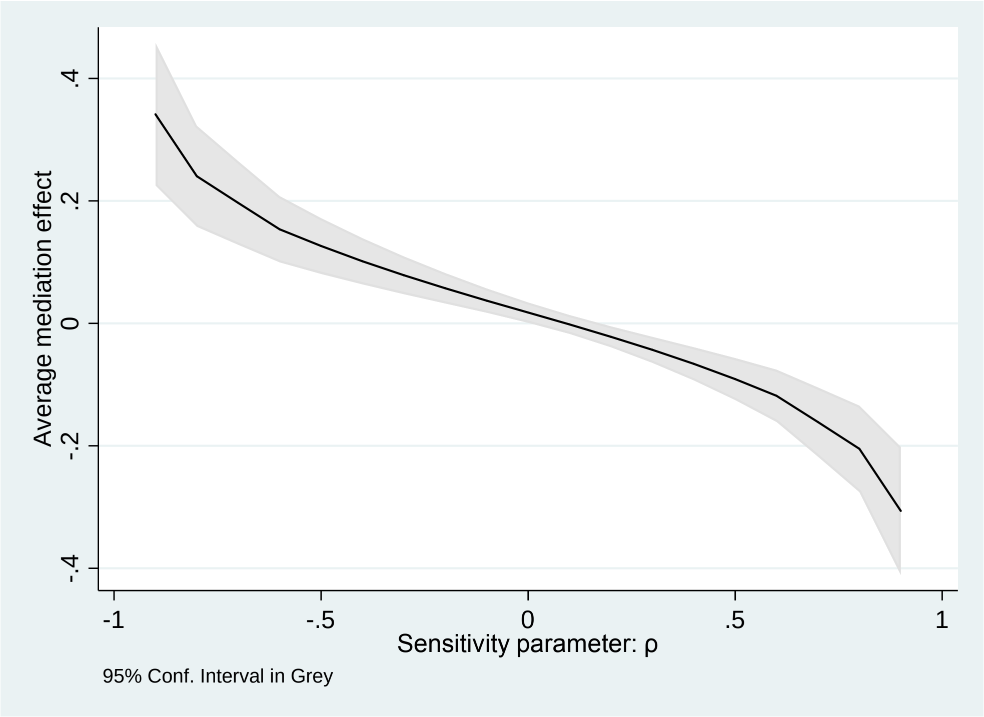

Panel B. Uganda

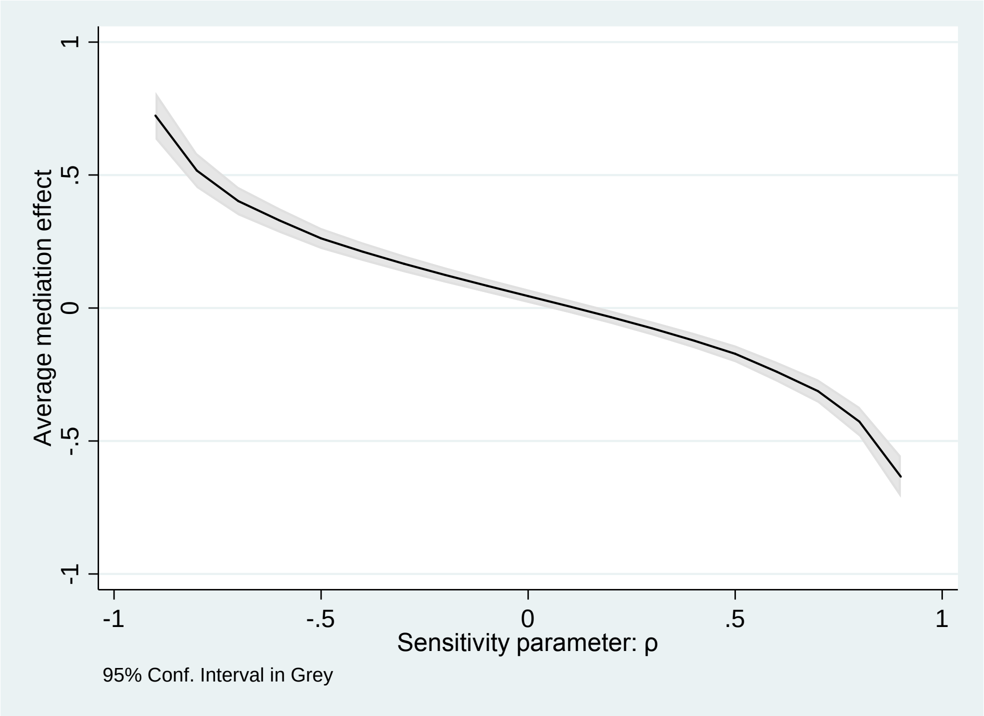

Appendix Figure S.1. Sensitivity Analysis, using mother's knowledge of OFSP as a vitamin A source as mediator variable and adoption as the outcome variable, including interaction terms, Mozambique and Uganda

Panel A. Mozambique

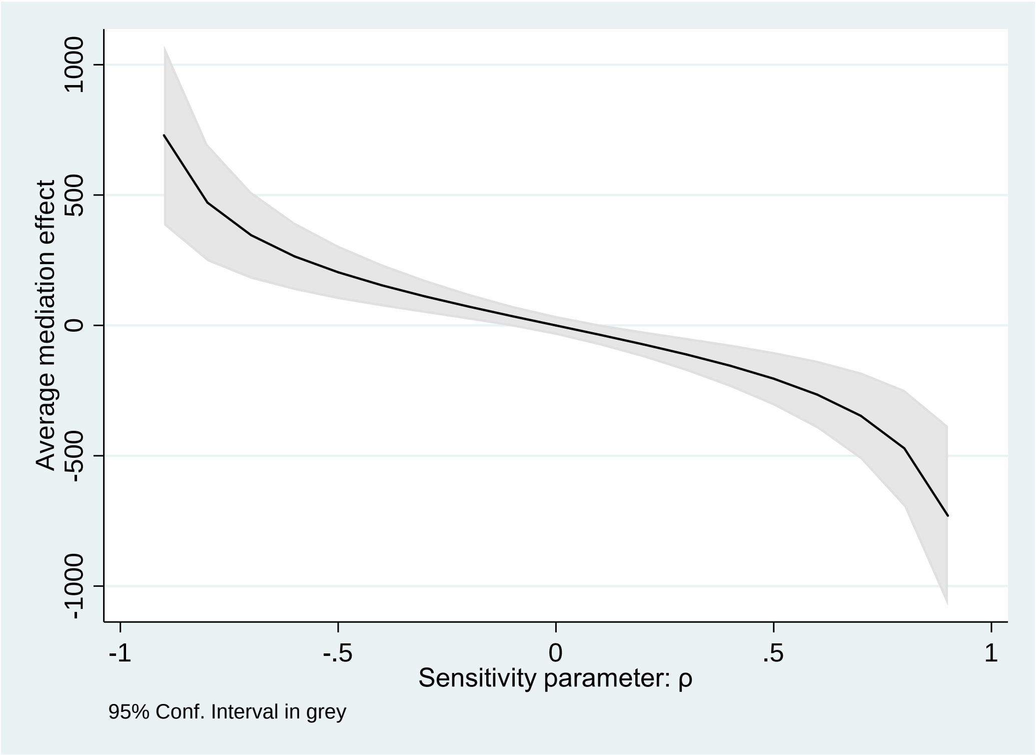

Panel B. Uganda

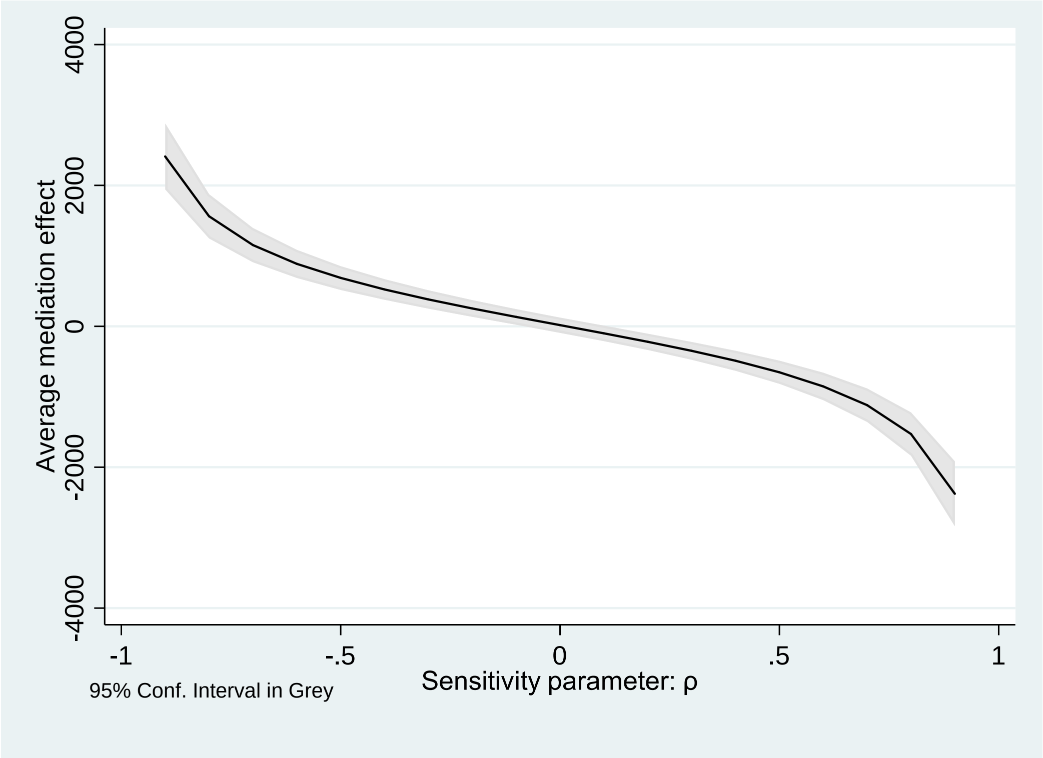

Appendix Figure S.2. Sensitivity Analysis, using mother's knowledge of OFSP as a vitamin A source as mediator variable, for vitamin A intakes among reference children as the outcome variable, including interaction terms, Mozambique and Uganda

Supplementary Tables

Appendix Table S.1. p-values for Wild bootstrap models, direct effects of treatment on outcomes, Mozambique

	Separate Treatment Groups		Combined Treatment Group
Dependent Variable:	Model 1	Model 2	
Adoption Indicator	<0.001	<0.001	<0.001
Share of OFSP in SP Area	<0.001	<0.001	<0.001
Total Area, OSP (Acres)	<0.001	<0.001	<0.001
Knows OFSP a source of vitamin A	<0.001	0.016	<0.001
Number of Messages Known	0.002	<0.001	<0.001
Vitamin A (mcg RAE)	<0.001	0.008	<0.001

Notes: Each row includes two separate regressions, one in which the treatment groups are separate and one in which they are combined. P-values are based on 1000 replications of a wild cluster bootstrap (Cameron, Gelbach, and Miller, 2008).

Appendix Table S.2. Lee Bounds estimates for treatment effects, discrete adoption and nutrition knowledge indicators

	Mozambique	Uganda
Growing OFSP at endline?		
Lower Bound	0.674	0.607
	(0.028)	(0.025)
Upper Bound	0.683	0.619
	(0.034)	(0.028)
95% Confidence Interval	[0.631, 0.747]	[0.562, 0.669]
Knows OFSP is a source of vitamin A		
Lower Bound	0.292	0.441
	(0.044)	(0.028)
Upper Bound	0.301	0.451
	(0.047)	(0.031)
95% Confidence Interval	[0.211,0.389]	[0.390,0.512]
Number of Vitamin A Facts Known		
Lower Bound	0.458	0.555
	(0.059)	(0.052)
Upper Bound	0.487	0.592
	(0.054)	(0.059)
95% Confidence Interval	[0.353,0.583]	[0.476,0.708]

Notes: Standard errors on bounds bootstrapped using 100 replications. Only discrete variables can be used to tighten bounds, so in Mozambique, the two significant correlates of attrition, female headship and access to lowlands, were included to tighten bounds. In both countries, for the "Knows OFSP is a source of vitamin A" variable we use the baseline value of whether the mother indicated OFSP as a vitamin A source to tighten bounds.

Appendix Table S.3. Impacts of REU Models 1 and 2 on measures of adoption at endline, adjusted for attrition in Uganda

	Uganda					
	Adopted OFSP		Share of OFSP in SP Area		Total Area, OFSP	
	(1)	(2)	(3)	(4)	(5)	(6)
**Panel A: Model 1 versus Model 2**						
Model 1	0.616***	0.630***	0.438***	0.426***	0.169***	0.170***
	(0.040)	(0.030)	(0.027)	(0.023)	(0.018)	(0.016)
Model 2	0.579***	0.601***	0.415***	0.409***	0.113***	0.118***
	(0.071)	(0.040)	(0.039)	(0.040)	(0.015)	(0.014)
Additional Covariates?	No	Yes	No	Yes	No	Yes
Test H_0: Model 1 = Model 2 (p-value)	0.658	0.563	0.618	0.692	0.016	0.018
**Panel B: Average treatment effect of both interventions**						
Treated	0.606***	0.622***	0.432***	0.422***	0.154***	0.157***
	(0.034)	(0.025)	(0.023)	(0.020)	(0.014)	(0.013)
Additional Covariates?	No	Yes	No	Yes	No	Yes
Endline Control Mean	0.054		0.027		0.008	
Number of obs.	975	975	751	751	975	975

Notes: All models are single difference models at endline. Baseline levels of adoption and area planted with OSP were very low, and so were omitted from these models. The share of OFSP in SP area has 224 missing observations in Uganda because these households did not grow any sweet potato. Tests of equality of impact of Model 1 and Model 2 are adjusted Wald tests. Average treatment effects reported at the bottom of the table are average impacts over Model 1 and Model 2, using the same specification for that column in a separate regression. Additional covariates included in some specifications, all measured at baseline, are whether or not the household had access to off-farm work, the number of male and female adults, whether the household head was male, whether or not a nutrition promoter lived in the household, whether the household grew sweet potato in 2006, and per-capita expenditures. All regressions include strata level fixed effects. Standard errors are clustered at the farmer group level in Uganda. *** significant at the 1 percent level.

Source: Uganda baseline and endline surveys, REU project.

Appendix Table S.4. Impacts of REU Models 1 and 2 on nutritional knowledge indicators at endline, adjusted for attrition in Uganda

	Uganda			
	Knows OFSP a source of vitamin A, 2009		Number of vitamin A Facts Known, 2009	
**Variable**	**(5)**	**(6)**	**(7)**	**(8)**
**Panel A: Model 1 versus Model 2**				
Model 1	0.459***	0.465***	0.555***	0.563***
	(0.040)	(0.030)	(0.063)	(0.062)
Model 2	0.445***	0.456***	0.605***	0.618***
	(0.059)	(0.039)	(0.114)	(0.107)
Additional Covariates?	No	Yes	No	Yes
Test H_0: Model 1 = Model 2 (p-value)	0.827	0.831	0.684	0.631
**Panel B: Average treatment effect of both interventions**				
Treated	0.456***	0.462***	0.568***	0.577***
	(0.035)	(0.028)	(0.058)	(0.057)
Additional Covariates?	No	Yes	No	Yes
Endline Control Mean	0.258		0.856	
Number of obs.	975	975	975	975

Notes: Regressions are ANCOVA models controlling for baseline level of the outcome. Tests of equality of impact of Model 1 and Model 2 are adjusted Wald tests. Average treatment effects reported at the bottom of the table are average impacts over Model 1 and Model 2, using the same specification for that column in a separate regression. Additional covariates are listed in the notes for Table 4. All regressions include strata level fixed effects. Standard errors are clustered the farmer group level in Uganda. *** Significant at the 1 percent level; ** significant at the 5 percent level; * significant at the 10 percent level.
Source: Uganda baseline and endline surveys, REU project.

Appendix Table S.5. Average Impacts of REU on Share of OFSP in Sweet Potato Area at Endline, Including Nutrition Knowledge Mediating Variables, and Estimates of ACME and ADE for the Role of Nutrition Knowledge in OFSP Adoption, Mozambique and Uganda

	Mozambique				Uganda			
	(1)	(2)	(3)	(4)	(5)	(6)	(7)	(8)
**Panel A: Coefficient Estimates**								
Treated	0.609***	0.590***	0.609***	0.593***	0.438***	0.413***	0.423***	0.411***
	(0.036)	(0.032)	(0.040)	(0.035)	(0.029)	(0.024)	(0.025)	(0.022)
Knows OFSP is source of vitamin A, endline	0.092***	0.057*			-0.013	0.020		
	(0.031)	(0.033)			(0.026)	(0.024)		
Number of vitamin A facts known, endline			0.051**	0.025			0.014	0.020*
			(0.022)	(0.022)			(0.014)	(0.012)
Additional Covariates?	No	Yes	No	Yes	No	Yes	No	Yes
Number of Obs.	534	534	534	533	751	751	751	751
R^2	0.488	0.514	0.485	0.511	0.396	0.450	0.396	0.451
**Panel B: Estimate of ACME and ADE**								
Treatment effect on knowledge	0.295	0.244	0.467	0.348	0.452	0.454	0.567	0.573
Knowledge effect on adoption	0.092	0.056	0.051	0.025	-0.013	0.020	0.014	0.020
ACME	0.027***	0.014**	0.022*	0.009	-0.006	0.009	0.008	0.012*
	(0.009)	(0.007)	(0.012)	(0.008)	(0.012)	(0.011)	(0.008)	(0.007)
ADE	0.610***	0.589***	0.614***	0.594***	0.438***	0.423***	0.424***	0.420***
	(0.035)	(0.032)	(0.038)	(0.034)	(0.028)	(0.026)	(0.025)	(0.024)
Share of Treatment Effect, Vitamin A Messages	4.2	2.3	3.5	1.4	1.4	2.1	1.8	2.8
Correlation, residuals	<0.0001	<0.0001	<0.0001	<0.0001	0.0064	0.0039	-0.0012	0.0012

Notes: Standard errors are clustered at the village level in Mozambique and the farmer group level in Uganda Additional covariates are listed in the notes for Table 4. All regressions include strata level fixed effects. *** Significant at the 1 percent level; ** significant at the 5 percent level; * significant at the 10 percent level.

Source: Mozambique and Uganda baseline and endline surveys, REU project.

Appendix Table S.6. Average Impacts of REU on Adoption at Endline, Including Nutrition Knowledge Mediating Variables, adjusted for attrition in Uganda

	Uganda			
	(5)	(6)	(7)	(8)
**Discrete Measure of OFSP Adoption**				
Treated	0.531***	0.576***	0.576***	0.600***
	(0.037)	(0.028)	(0.036)	(0.026)
Knows OFSP is source of vitamin A, endline	0.163***	0.100***		
	(0.031)	(0.031)		
Number of vitamin A facts known, endline			0.053***	0.039**
			(0.017)	(0.015)
Additional Covariates?	No	Yes	No	Yes
Number of Obs.	975	975	975	975
R^2	0.410	0.469	0.395	0.464
**Share of OFSP in Sweet Potato Area**				
Treated	0.437***	0.411***	0.424***	0.410***
	(0.028)	(0.024)	(0.025)	(0.021)
Knows OFSP is source of vitamin A, endline	-0.009	0.020		
	(0.025)	(0.023)		
Number of vitamin A messages known, endline			0.014	0.019*
			(0.013)	(0.011)
Additional Covariates?	No	Yes	No	Yes
Number of Obs.	751	751	751	751
R^2	0.417	0.468	0.418	0.469

Notes: Standard errors are clustered at the farmer group level in Uganda Additional covariates are listed in the notes for Table 4. All regressions include strata level fixed effects. *** Significant at the 1 percent level; ** significant at the 5 percent level; * significant at the 10 percent level.
Source: Uganda baseline and endline surveys, REU project.

Appendix Table S.7. P-values for Wild Bootstrap models, Equations including Mediators, Mozambique

	Dependent Variable:			
Mediating Variable:	Adoption	Adoption	Vitamin A	Vitamin A
	(1)	(2)	(3)	(4)
Treatment Effect	<0.001	<0.001	0.492	0.582
Adoption Indicator			0.006	0.002
Knows OFSP a source of vitamin A	0.046			0.492
Number of Messages Known		<0.001	0.696	

Notes: Each column includes a specific regression. P-values are based on 1000 replications of a wild cluster bootstrap (Cameron, Gelbach, and Miller, 2008).
